# NetCapDB: measuring bioinformatics capacity development in Africa

**DOI:** 10.1186/s13104-016-1950-5

**Published:** 2016-03-05

**Authors:** Hocine Bendou, Jean-Baka Domelevo Entfellner, Peter van Heusden, Junaid Gamieldien, Nicki Tiffin, for the H3ABioNet Consortium, as members of the H3Africa Consortium

**Affiliations:** South African National Bioinformatics Institute/Medical Research Council of South Africa Bioinformatics Unit, University of the Western Cape, Cape Town, South Africa

**Keywords:** Bioinformatics, Africa, Capacity, Monitoring and evaluation, Capacity development

## Abstract

**Background:**

The National Institutes of Health (USA) has committed 5 years of funding to the Bioinformatics Network of the Human Heredity and Health in Africa initiative. This pan-African network aims to develop capacity for bioinformatics research, in order to provide support to human health genomics research programs ongoing on the continent. Over the 5 years of funding, it is imperative to track changes in bioinformatics capacity at the funded centres and to document how the funding has translated into capacity development during this time frame.

**Results:**

The Network capacity database, NetCapDB, is a relational database that captures quantitative metrics for bioinformatics capacity, and tracks the changes in these metrics over time. A graphical user interface allows for straight-forward, browser-based data entry by users across Africa; and for visual and graph-based exploration of captured data. A reporting interface allows for semi-automated generation of standardized reports for monitoring and evaluation purposes.

**Electronic supplementary material:**

The online version of this article (doi:10.1186/s13104-016-1950-5) contains supplementary material, which is available to authorized users.

## Background

The Human Heredity and Health in Africa (H3Africa) programme was established in 2010 to facilitate research into the genetics and environmental determinants underlying disease in Africa [1], and is funded by the Wellcome Trust (UK) and the National Institutes of Health (NIH, USA). Within this programme, the NIH funds the H3Africa Bioinformatics Network (H3ABioNet, http://www.h3abionet.org, [2]) to build capacity for bioinformatics research on the African continent. Currently, 32 network nodes are distributed across 15 African countries, with two additional nodes based in North America and the United Kingdom. H3ABioNet funding supports bioinformatics training and research, as well as the development of computational infrastructure including improved data storage, server capacity and internet bandwidth.

In order to report objectively on the development of bioinformatics capacity in African H3ABioNet member institutions, we have defined metrics to assess bioinformatics capacity, and have developed the network capacity database (NetCapDB) to capture changes in key areas that reflect the increasing research, training and infrastructural capabilities of H3ABioNet members over the funded period.

The primary outcome to be assessed is the conceptual construct defined as bioinformatics capacity development. In order to measure capacity, we defined a series of conceptual variables relating to research activity, education and training, skills development, and computational infrastructure. For each of these we further defined a series of operational variables that can be directly and objectively measured, and captured in NetCapDB (Table 1). To be able to assess changes in bioinformatics capacity that reflect *capacity development over time*, we included a time element that allows capture of the same metrics at specified time intervals that match the periods for reporting progress to the funders. Thus changes in defined metrics can be visualised across the specified time periods to indicate changes occurring over time. Finally, we have developed an interface for automated reporting of key metrics at specified time intervals, to ensure consistent, accurate and easily generated reports for stakeholders. This can assist with rapidly identifying key areas where translation of funding into bioinformatics capacity is particularly successful, or is underperforming. Consistent reporting using operational variables that can be objectively measured allows for clear tracking of change in bioinformatics capacity across Africa, within the context of H3Africa funding for the H3ABioNet initiative.

**Table 1:** Categories and structure for data capture

Main categories	Conceptual variables	Operational variables
Research capacity	Scientific productivity	Number of peer-reviewed papers, node-centric and per individual.
	Scientific communication	Number of invited talks, plenary lectures
		Number of oral presentations at international conferences
		Number of posters at international conferences
Personnel capacity	Academic capacity	Listing of academic node members with qualifications, affiliations
	IT resources	Listing of IT personnel
	Skills capacity	Listing of skills, per individual
Collaboration	Involvement in H3ABioNet	Project involvement, node-centric and per individual
	H3ABioNet working group activity	H3ABioNet working group involvement per individual
	Involvement in H3Africa research projects	Listing of H3Africa project participation
	H3Africa active engagement	H3Africa working group involvement per individual
	Within Africa collaboration	Travel undertaken, origin and destination
		Number of co-authorships with other African Scientists
		Number of collaborative projects ongoing, African and other
	Professional engagement	List of professional society membership, per individual
Additional funding	Sustainability and increased capacity for funding leverage	Sources of personnel funding other than H3ABioNet
		Sources of research funding other than H3ABioNet
		Grants currently held, per individual and per node
		Funding sources, per individual and per node
Education and training	Training of students	Number of M.Sc and Ph.D students currently registered, supervisors
		Number of M.Sc and Ph.D students graduating annually
	Staff training activities	Courses taught (person-days), node-centric and per individual
		Courses attended (person-days) per individual
	Hosting training and workshops	Courses hosted
Infrastructure	Quality of internet access	Up/down bandwidth
	Videoconferencing facilities	Videoconferencing equipment
	Computing power	Number and type of cores and associated RAM
	Equipment	Number of computers, laptops and terminals
	In-house training facilities	Numbers, size and facilities of classrooms
		Number of training workstations

## Methods

**Fig. 1:**
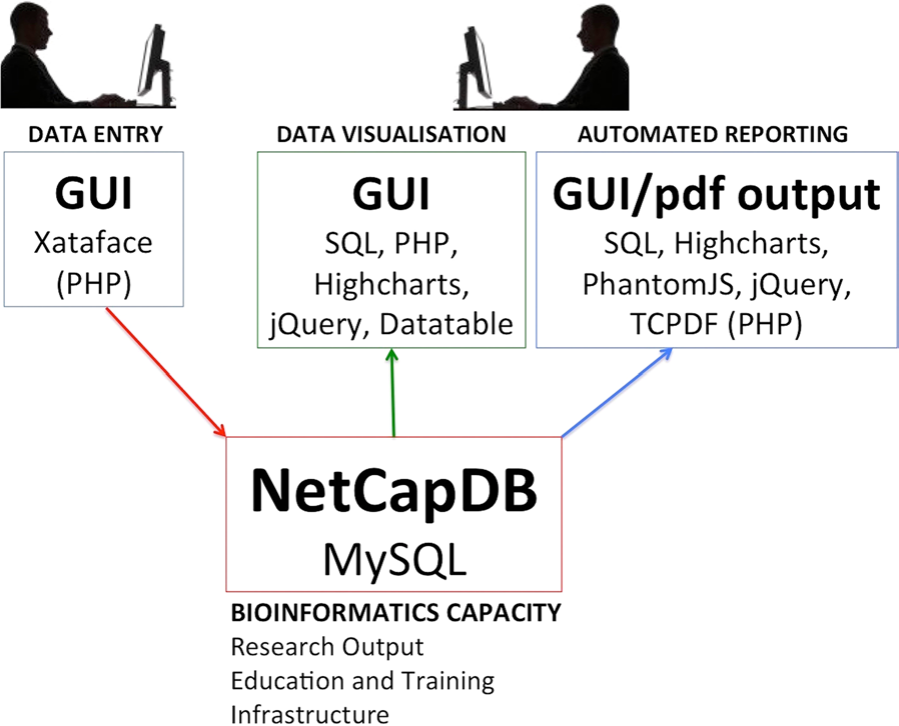
Schema showing software used for data entry, querying and semi-automated report generation

An overview of the functionality of NetCapDB is shown in Fig. 1.

### Security protocols

The NetCapDB database has been created and is stored on a virtual machine (VM) on the SANBI network. The operating system for the VM is Ubuntu 12.04, whose official support is guaranteed until 26th of April 2017. Access to the VM from outside the SANBI network is restricted by firewall so that only the web server is accessible. Within the SANBI network, login to the NetCapDB VM is restricted to only SANBI and NetCapDB administrators, who may log in using password authentication. Furthermore, root access is restricted to only SANBI and NetCapDB administrators. The web interface restricts data at the node level, so that each node has its own access, secured using a login and password, and cannot view the information entered by other nodes; except some global tables in use wherever data has to be shared across nodes to avoid redundancy and to ensure consistency—see below.

### The graphical user interface

**Fig. 2:**
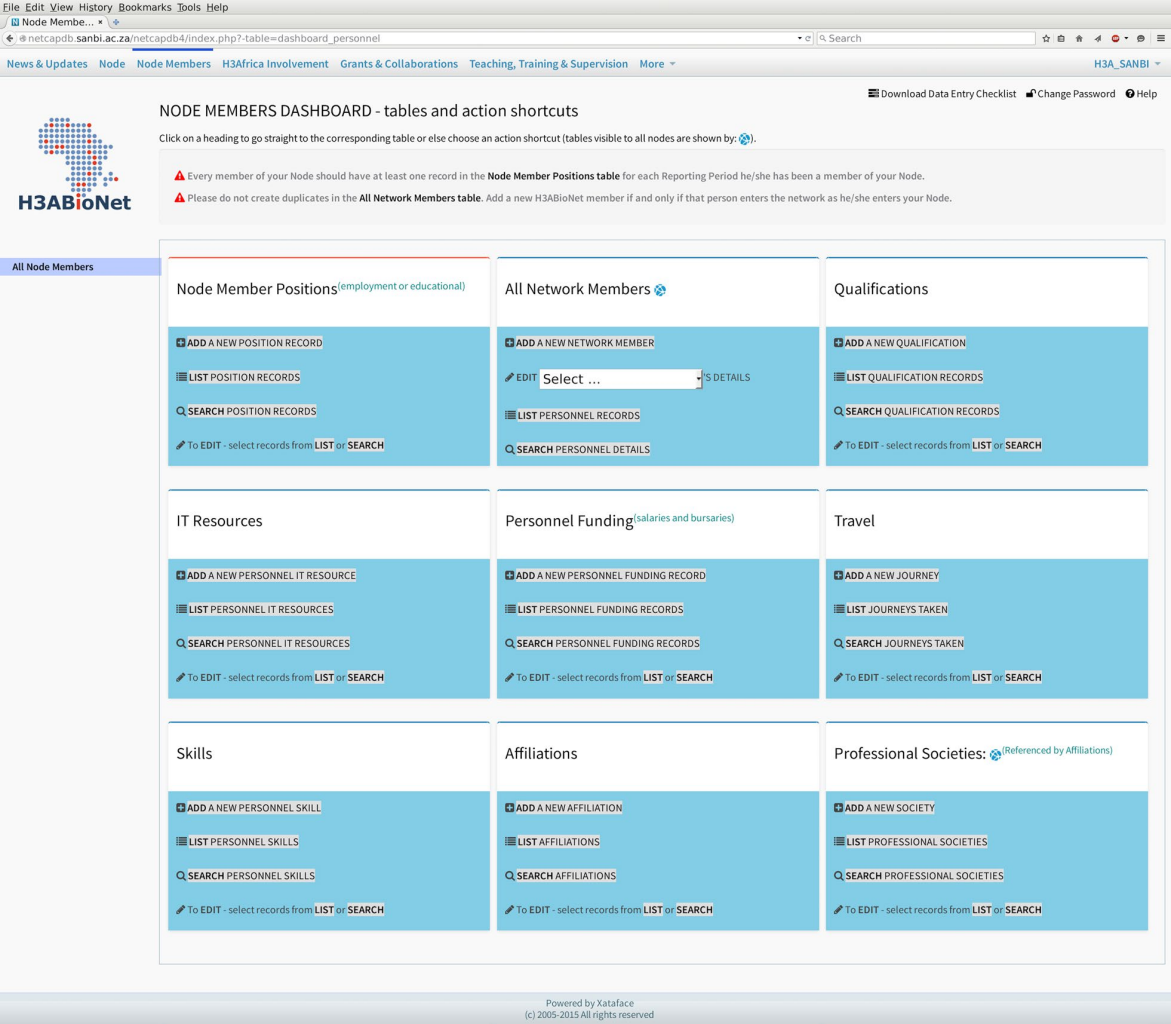
Screenshot showing the captured metrics for research output and publications

**Fig. 3:**
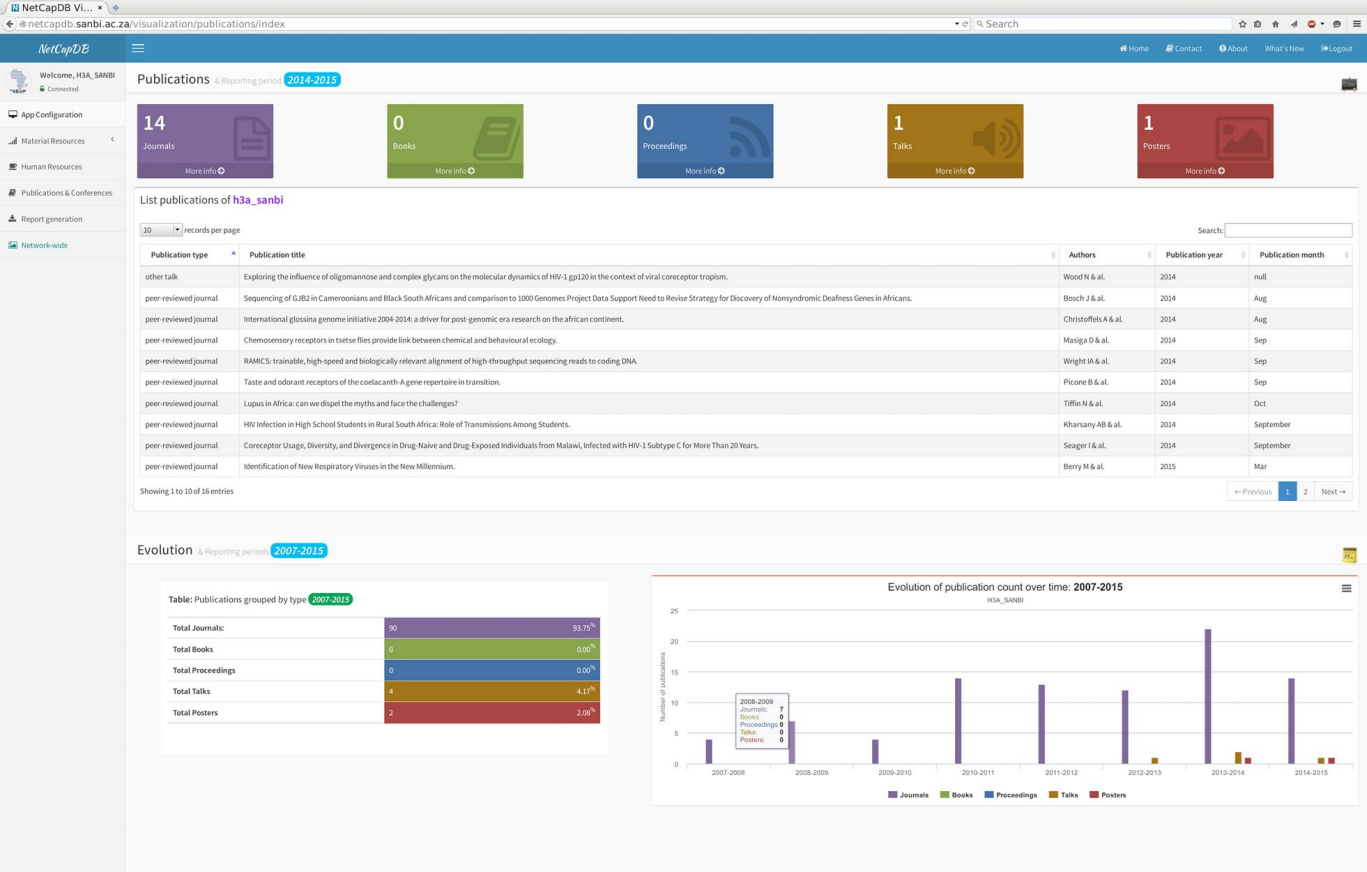
Screenshot showing the dashboard for navigating data-capture within the different categories

Xataface is an open source tool written in PHP, and was used as the foundation of the graphical user interface (GUI) to facilitate interaction with the NetCapDB database, allowing users to update, delete and search for data with a user-friendly browser-based front end (Xataface version 2.0.3, written by S. Hannah and available from http://www.xataface.com, 2013). Xataface uses structured configuration files, or INI files, and provides multiple HTML templates that are ready to use, for example to view the records of a table or to add a new record (Fig. 2). In the *tables* section of the ‘conf.ini’ file, as recommended in the Xataface documentation, we declared the 30 important table names as properties, with a text name assigned as a value to each table name in order to assist with efficient table referencing through descriptive table naming. In addition we added seven other dummy-table properties to the *tables* section. These properties are filtered by the PHP module ‘ApplicationDelegate.php’ for use in the navigation menu of the application. Each menu item is linked to one of the seven dashboard HTML files, allowing users to browse the tables in an orderly and categorized manner (Fig. 3).

### Database structure

The relational tables in the NetCapDB database are stored using MySQL, an open source database management system frequently used for web applications (http://www.mysql.com/). In total, there are 128 tables. Of these tables, 35 are created automatically by Xataface and store Xataface information. From the 30 primary tables, one is used to store user data, including login and encrypted password and is only accessible to the database administrators, and 29 tables are divided into seven dashboards that are presented in the GUI section for data entry by the nodes. Nine tables store information for the Dashboard menus. Tables are either core tables that contain data without a ‘reporting period’ component, or dynamic tables that record data reporting period-specific. Controlled vocabularies for drop-down lists are also stored in defined tables; and every modification or change made to stored data, as well as the database itself, is logged in 30 “change” tables, that allow for tracking changes to data that have been entered previously, should recovery be necessary. Finally, seven dummy tables are created for the dashboard menus.

Not all data are specific to one node. If users enter a novel item for such a field, it is added to a global table, accessible to all users at all nodes, so that it does not need to be re-entered for further reporting. In those cases where a field entered might be used in multiple entries, the global table collates all the drop-down options for the field, which can be viewed and updated by all nodes. Thus, for example, conferences attended by many network members only need to be manually entered for the first reporting entry, and can be selected from the global table by subsequent users at all nodes who wish to report attending the same conference.

Sets of permissions can be associated with user profiles through definitions in the ‘permissions.ini’ file. These user profiles are associated with sets of tables, allowing fine-grained control over operations permitted to the associated users. Permissions are operation names e.g., *list, edit, copy,* etc. bearing Boolean values. If the property has a value of 0 (e.g., *copy* = *0*), the operation (copy) and other related actions (e.g., copy set) defined in ‘actions.ini’ are prohibited: in our example this will hide the *copy* button from the list form, and if present it allows duplication of one or more records in the table. The copy feature is an example of one of the more powerful features of Xataface that we have harnessed to avoid the need to manually re-enter data. Especially when starting a new reporting period, re-entering existing data can be tedious, but in NetCapDB most of the tables contain the reporting period as a field, and the copy operation allows semi-automated copying and updating of records into a new reporting period.

### Metrics

**Fig. 4:**
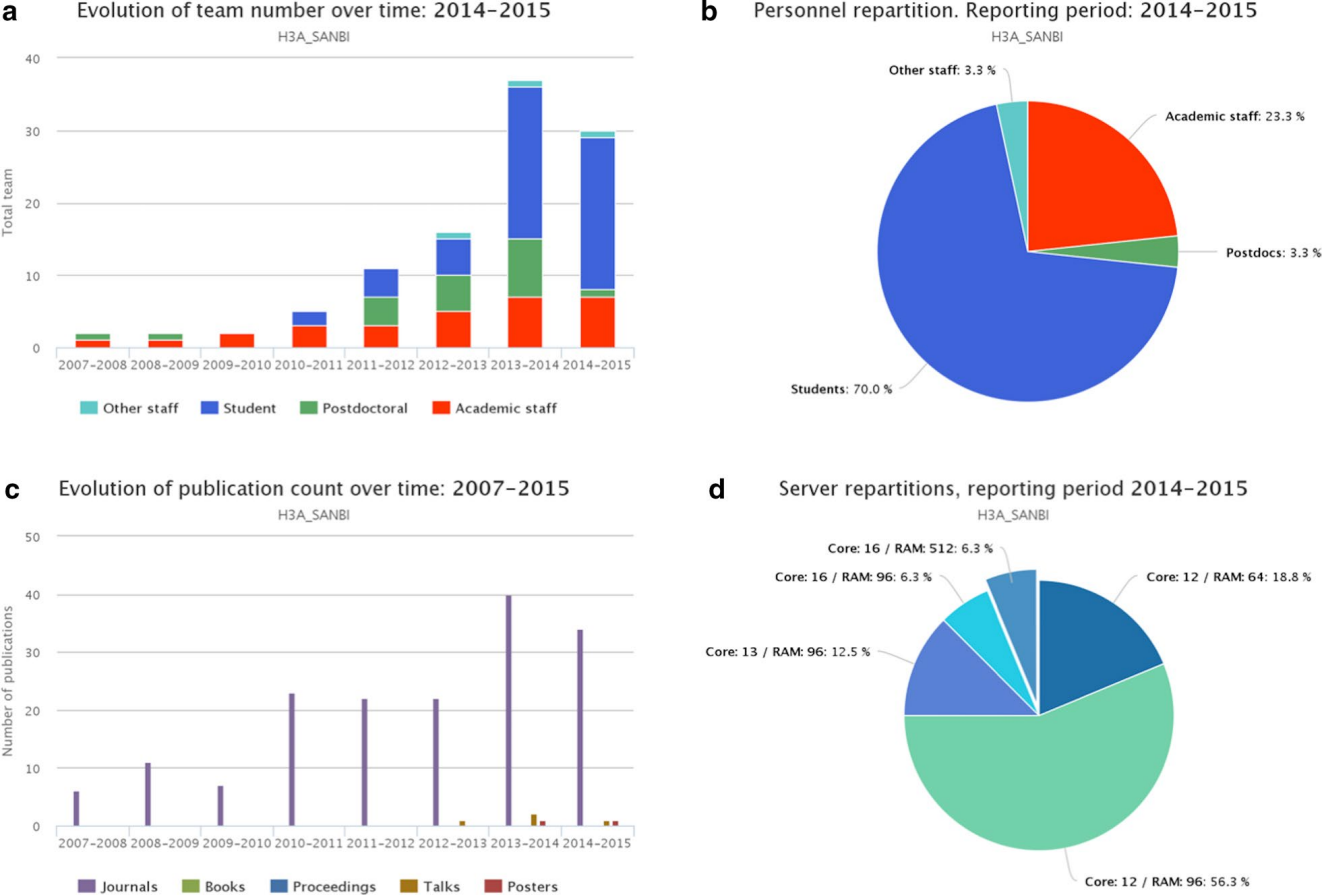
Example of some metrics collected for the SANBI node at the University of the Western Cape, South Africa, up to the reporting period ending in June 2015. **a** Number and type of personnel at node from 2007 to 2015; **b** detailed view of personnel in reporting period 2014–1015; **c** count of publications and scientific communications from 2007 to 2015, and **d** detailed server capacity in 2015

The measures chosen for evaluation were determined according to the specific capacity-building objectives of the funded project defined in the approved funding proposal. At a conceptual level, metrics captured by NetCapDB include training and capacity development, networking and communication, and research. Specific metrics collected reflect those commonly in use for research assessment, including: number of peer-reviewed publications from individual nodes as well as from collaborative projects within the network and the H3Africa Consortium, conference presentations, network members and graduates trained, network member participation as trainers, within-Africa research collaboration and funding external to H3ABioNet raised by network members. Ongoing assessment of metrics evaluated is also provided by the Scientific Advisory Board (Fig. 4).

The reporting entities for the database are the research units, or H3ABioNet nodes. These are based at separate institutions and there may be multiple nodes per country. Currently, 32 H3ABioNet nodes are registered as members of the network. A significant challenge has been the large diversity in prior capacity between bioinformatics units across the continent, and for this reason change is tracked in individual nodes over time rather than measuring achievements in absolute numbers. This ambitious scope of the project has necessitated restricting the complexity of metrics captured, because of African-specific challenges to bioinformatics research. Key features have been establishing core computing infrastructure, data storage servers and trained systems administrators, as well as sufficient bandwidth or workarounds to accommodate extremely poor connectivity in some regions. Node-centric data that are captured include: the computational capacity of the node with the number and specifications of servers (CPU and RAM), the type of Internet link and the corresponding upload/download bandwidth, video-conferencing facilities, the number of classrooms and teaching workstations. The *servers*, *bandwidth* and *classrooms* tables are created to store these data.

Other challenges include the retention of locally trained bioinformaticians on the continent and developing pan-African collaborative research projects in a shift away from the existing paradigm of externally-driven research programs. There is a separate record in the *Node members* table for every individual involved in H3ABioNet. Each member has held one or many positions in the node, and has obtained one or several qualifications and skills in different fields. All these data are saved in *positions*, *qualifications* and *skills* tables, and changes in these metrics over time can be captured. There is also a global table of network members that is accessible to all users to avoid duplicate entries for individuals, records demographic data and allows for unambiguous identification of any H3ABioNet member. Research funding sources, professional society affiliations and institutional affiliations are all captured at the individual level. Frequency and destinations of travel are also recorded.

A central aim of the H3BioNet network is to foster collaborative bioinformatics research within Africa. NetCapDB collects information about H3Africa project involvement of individuals, as well as other collaborations ongoing. Information is captured about research funding as well as project outputs including peer-reviewed publications, and talks and posters presented at conferences. Publication metrics include all types of papers published, and are used to track increases in node-specific publications, collaborative publications between nodes and with other H3Africa projects, and within-Africa collaborations with researchers outside the H3Africa Consortium. This indicates increasing capacity by H3ABioNet nodes to contribute bioinformatics skills to research within Africa, to reduce the dependence of African researchers on off-continent bioinformatics research skills. Analysis of journals and titles can be used to identify bioinformatics-specific output. Furthermore, analyzing the network data pertaining to collaborations (within H3ABioNet and with other researchers on the continent), co-publications, travel and successful multiple-node funding applications will indicate individuals and nodes that are particularly crucial to the overall success and extension of bioinformatics capacity on the continent; as well as highlighting areas where more attention should be directed to fostering integrated and collaborative research.

Information about funding bodies is captured in a global table, and node-specific funding information indicates the ability of nodes to extend their resources outside of H3ABioNet funding. Extensive data about education are collected. Information about workshops and courses are captured in a global table *courses and workshops*, and participation is recorded for those who are teaching (As a trainer) as well as those attending the event (*As a trainee*). Supervision of students and student graduations are also captured. Students are recorded within the network members table to allow tracking of training/career advancement of individuals from the nodes. With each funded year, the researchers’ career progression, publication record, training and successfully funded grants indicate the increase as well as retention of skills on the continent; and by measuring the country of graduation of all network members the increasing number of African graduates and their retention within Africa can be tracked within the context of H3ABioNet funding.

### Data validation

Data validation is performed by MySQL triggers on update or insert when records are edited or added, as well as validation checks implemented in the user interface (Xataface). Assessment of reported metrics is undertaken annually through the H3ABioNet central administration and graphs and analyses that are built from the data allow for more advanced data-level checking ensuring consistency and accuracy. Internal working groups also engage with tracking progress within their specific domains.

The nodes provide annual reporting on capacity development metrics and renewal of node funding is reliant on realizing reporting requirements. Further, complete reporting is requisite for involvement and authorship in network capacity-related publications. Nodes have annual individual meetings with network administration to review progress and achievements, as well as participation and review at annual general meetings.

### Querying data

In order to query the database, SQL queries are implemented and executed in PHP using the PDO interface (PHP Data Objects: to access a database server) methods: prepare() and execute(). The prepare() function prepares an SQL statement in string format, to be executed by the execute() method; and returns a statement object (http://php.net/manual/en/index.php). If there are no errors in this process, the resulting set is retrieved in a PHP array using the fetchAll() method.

Wherever possible, nodes have entered key information for the 5 years preceding the starting date of H3ABioNet funding in order to provide a baseline comparison point for node-centric analysis. Currently, 3 years of funding data are available for analysis, and these data are also reported and assessed annually per node to track evolving capacity over the duration of the funding.

### Generating semi-automated reports

Multiple Javascript open source tools are used to create graphs in a suitable format for reporting purposes (JPEG, PNG). We selected the Highcharts tool (http://www.highcharts.com) for its compatibility with PhantomJS (http://phantomjs.org/) to export the figures to the SANBI server. The PHP array to be represented as figures is converted into JQuery (https://jquery.com/) array using the json_encode() function. To generate the PDF documents, we use TCPDF (http://www.tcpdf.org) which integrates free text fields entered by the user with the Highcharts figures exported by PhantomJs to the server.

The tutorial for NetCapDB is included as Additional file 1.

The H3ABioNet general assembly meets annually, and a significant portion of the meeting reviews data entry and report generation. Additionally, the online 3-monthly meetings of the general assembly provide a forum for feedback specifically about using NetCapDB. Independent oversight is also provided by the Scientific Advisory Board, who to date have given very positive feedback about NetCapDB functionality and metrics.

## Conclusions

To date, no pan-African bioinformatics capacity assessment has been undertaken; and the tracking of change in capacity by measuring well-recognised, quantitative metrics over time is also a novel development. Independent international organisations such as the Global Organisation for Bioinformatics Learning, Education and Training (GOBLET) [3, 4] provide networks for training and education, and the African Society for Bioinformatics and Computational Biology (ASBCB) fosters bioinformatics collaboration on the continent; but the provision of a substantial and encompassing grant specifically to invest in bioinformatics capacity in Africa is unprecedented.

NetCapDB successfully captures quantitative metrics that reflect bioinformatics capacity at nodes of the H3ABioNet (http://www.H3ABioNet.org), a member of the H3Africa Consortium (http://www.H3Africa.org). The database is designed to collect these metrics longitudinally for the duration of the funded programme and beyond. The conceptual variable “bioinformatics capacity” has been mapped clearly onto quantitative metrics, making it possible to measure the change over time within the defined domains of research infrastructure, research output and education/training. A user-friendly GUI facilitates data entry, with minimal repetition and manual input required; and the use of global tables provides a compromise between standardizing data entry and the ability to add new information becoming available over time—with the added incentive of greatly reduced data entry time. Automated generation of charts and tables, and semi-automated report generation per reporting period allow for efficient and accurate ongoing reporting within nodes, and to project funders and other stakeholders. This can also be used to identify areas of most and least bioinformatics capacity development per spend, to assist with funding decisions going forward.

One area that presented a significant challenge for data capture was the management of publication data. Automatic entry of data from a PubMed export was not always successful because of some ambiguities with data storage in the CSV PubMed output file. A preferable format for uploading PubMed publication records is the XML format, which is more verbose but provides cleaner publication data. Manual upload of publication data also leads to inconsistencies, and it would be preferable to incorporate a search engine of online databases that could present the user with an array of records from which to select and upload the appropriate publication. It would also be preferable to manage co-authorships within the network in an automated way, as the current system links every publication to a single author and can result in multiple records being stored for a single publication. We currently rely on our own postprocessing SQL scripts to establish coauthorships between several members of the network.

In general, feedback from the Scientific Advisory Board and users has been positive and the automated reporting facility has been used exclusively for an entire reporting period. Whilst the time required to enter requisite data in the first instance has been a stumbling point for some users, 6 monthly updates of existing records is less onerous.

Future directions for data collection include exploring the possibility of automated copying of appropriate information (for example, node members or infrastructure information) forward into the next reporting period to avoid the need to copy this information manually, as well as automated copy forward of information for taught courses that are repeated frequently. We will also monitor the impact of social networking on collaboration within the network and with research partners, through a recently launched twitter account at https://twitter.com/h3abionet.

Analysis of metrics generated by NetCapDB will be, by necessity, mostly descriptive of capacity change over the funded period. Whilst we can make some general network-wide conclusions about overall capacity change, we do not have a large enough sample base to make statistical inferences from the data. Furthermore, it would be unrealistic to attempt to measure statistical significance, because evolution of H3ABioNet as an entity and of the individual nodes is inevitably affected by a multitude of external factors that cannot necessarily be measured or standardized. The baseline from which each node is measured is also unique to each node. We therefore aim to compare data from the beginning of funding and where possible the preceding 5 years, to changing metrics over the funded period.

Building such an evolving picture over the 5 years, based on quantifiable and consistent metrics, affords the opportunity to assess capacity changes for each node on an ongoing basis. This allows identification of specific areas for each node where capacity expansion is marked over the funded period, as well as areas where growth in bioinformatics capacity is not being fully realized. This allows for data-informed modifications of approaches and funding streams throughout the course of the funding, ensuring maximal benefit per funding input: the metrics generated in this ongoing process will thus assist in organic tailoring of the network’s evolution to ensure successful outcomes. We believe that such a process of objective, ongoing evaluation of bioinformatics capacity building at all the H3ABioNet nodes will greatly improve the final achievements of the Consortium. Furthermore, the insights gained through the ongoing analysis of these metrics may assist in designing future capacity development programmes in this field.

Detailed information about the database schema and associated scripts is available from the authors on request.

## Additional file

10.1186/s13104-016-1950-5 NetCapDB v2 Data Entry Tutorial.

## Abbreviations

NIH: National Institutes of Health
H3Africa: Human Heredity and Health in Africa
H3ABioNet: H3Africa Bioinformatics Network
NetCapDB: Network Capacity Database
VM: Virtual Machine
GUI: Graphical User Interface
