# Supplementary material for: NetCapDB: measuring bioinformatics capacity development in Africa
Source: BMC Res Notes. 2016 Mar 5;9:144. doi: 10.1186/s13104-016-1950-5 (PMC4779554; doi:10.1186/s13104-016-1950-5)
Supplement: Supplementary file 1 — 10.1186/s13104-016-1950-5 NetCapDB v2 Data Entry Tutorial. [file 13104_2016_1950_MOESM1_ESM.pdf]

NetCapDB v2 Data Entry Tutorial

Jean-Baka Domelevo Entfellner, Hocine Bendou, Nicki Tiffin
(South African National Bioinformatics Institute, UWC, Cape Town)

May 21, 2015

Contents

1 Introduction, general remarks and guidelines 2
1.1 Dashboards . . . 3
1.2 Time-relatedness in NetCapDB . . . 3
1.3 Browsing tables . . . 3
1.4 Adding new records in NetCapDB . . . 3

2 Data Entry Tutorial, dashboard-wise 4
2.1 Node dashboard . . . 4
2.2 Node Members dashboard . . . 5
2.2.1 All Network Members . . . 5
2.2.2 Node Member Positions . . . 6
2.2.3 Personnel Funding . . . 6
2.2.4 Travels . . . 6
2.3 H3Africa Involvement dashboard . . . 7
2.4 Grants & Collaborations dashboard . . . 7
2.5 Teaching, Training & Supervision dashboard . . . 8
2.5.1 As a trainer . . . 8
2.5.2 As a trainee . . . 9
2.5.3 Student Supervision . . . 9
2.6 Publications & Conferences dashboard . . . 9
2.6.1 Publication Details . . . 10
2.6.2 Conference Attendees . . . 11
2.7 Reporting Periods dashboard . . . 11

1 Introduction, general remarks and guidelines

NetCapDB (`http://netcapdb.sanbi.ac.za`) is intended at capturing a trustworthy picture of the developmental status of the Nodes operating within the H3ABioNet network. It collects data about the Nodes' publications, their training and student supervision activities, but also some information regarding the Node personnel and infrastructure. A new version of the data entry interface has recently been implemented at SANBI (University of the Western Cape, Cape Town, South Africa) by Hocine Bendou, software developer, and Jean-Baka Domelevo Entfellner, postdoctoral fellow. This is accessible as usual through the "Data Collection" button from the URL cited above (see figure 1). This new version comes with some changes in the structure of the underlying database, with a very few new fields that you have to fill in. The SANBI team has also developed scripts which automatically build and display synthetic reports calculated on the data captured. A limited but growing set of Node-centric graphical data representations is already made available to you through the "Data Exploration" button from `http://netcapdb.sanbi.ac.za/`.

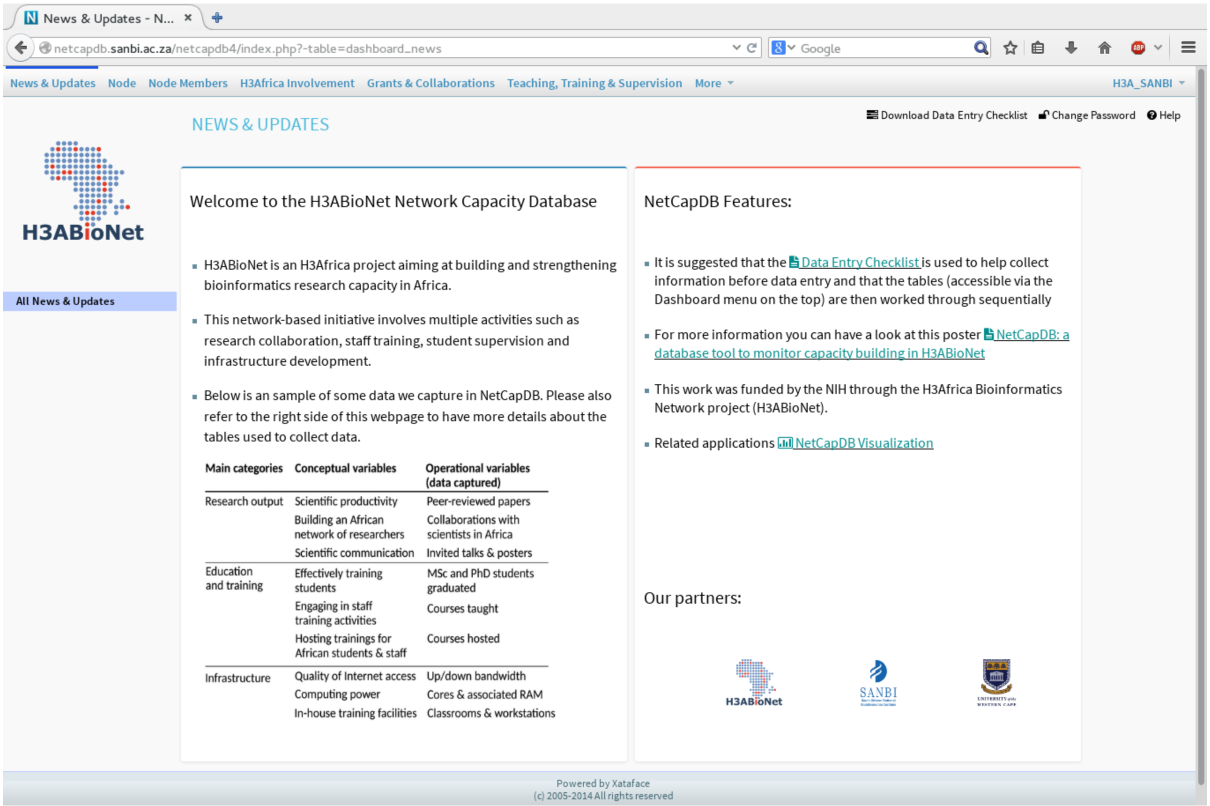

Figure 1: Homepage of the new NetCapDB data entry online interface, after user authentication.

Network-wide reports are also built from the data captured in the database, for use by the Central Node (UCT) and the H3ABioNet Scientific Advisory Board and Management Committe. Thus it must be clearly understood by all the H3ABioNet PIs that **the data you capture (or fail to capture) *will* have a major impact on the way your Node will be evaluated by the funders of the H3ABioNet project**.

1.1 Dashboards

The NetCapDB data entry website is divided into “dashboards”, each dashboard grouping several data tables. From anywhere within the data entry tool, you may jump to any dashboard by clicking on one of the corresponding buttons on the top menu (horizontal bar on top of the screen on figure 1). You may have to click on the *More* link to access the last two dashboards, *Publications and Conferences* and *Time Reference*. In this brief tutorial, we are going to walk you through the major steps leading to a complete and successful data entry. After some indications that hold for all the data entry tool, the present tutorial will logically be divided according to the different dashboards of the data entry interface.

1.2 Time-relatedness in NetCapDB

Key to NetCapDB is the concept of “Reporting Periods”. A Reporting Period is a one-year period starting on August 1st and ending on July 31st of the following year. This timespan corresponds to an NIH reporting year. Most data captured into NetCapDB are associated to a Reporting Period, for instance a personnel’s position, data concerning the quality of Internet access from a Node, grants secured by a Node, Node Member travels, etc.

Some data are static, which means they are unrelated to any Reporting Period. These are the data concerning personnel details (global table “All Network Members” under the “Node Members” dashboard).

Finally, journal articles and other written publications (posters, book chapters, etc) are associated with a year and a month of publication, so this is very important that you fill in these two fields when entering a publication manually.

1.3 Browsing tables

You may use the “Previous Table” and “Next Table” blue buttons on top of each table view in order to go to the previous or to the next table in the same dashboard, in a looping fashion.

Whenever you are listing the contents of a table, you may click on any column header in order to list the records according to that field, either by increasing order or, after a second click, by decreasing order.

1.4 Adding new records in NetCapDB

Every table in the database is made of a certain number of “records”, or “lines”. Each record contains several “fields”, that you are going to fill in through text boxes, drop-down selection lists, radio buttons or tickboxes.

To add a new record, whichever the table under consider, one has several options. We describe below the steps to be followed from the “List” view of any table:

- add a new record “from scratch”, using the “New record” button in the left margin. This will create a brand new record with empty fields that you are going to fill in.

- copy the information from a pre-existing record, updating zero (exact copy) or one of the fields (new record derived from the previous one). This operation creates a new record whose content is partially or totally copied from the original record. To do this, you first have to select one or several records from the table list view, and then click on the button “Copy” repeated on top and at the bottom of the table. Note that you can copy several records at the same time: for instance, in the “Node Member Positions” table under the Node Member dashboard, if several of your staff members have retained the same positions as in the last Reporting Period, you can simply copy the corresponding records, altering the field corresponding to the Reporting Period.

- bulk upload a series of publications from the PubMed import tool, to add at once several records in the “Publications” table. Of course this method is only valid for that specific table.

2 Data Entry Tutorial, dashboard-wise

2.1 Node dashboard

This dashboard in pictured in figure 2.

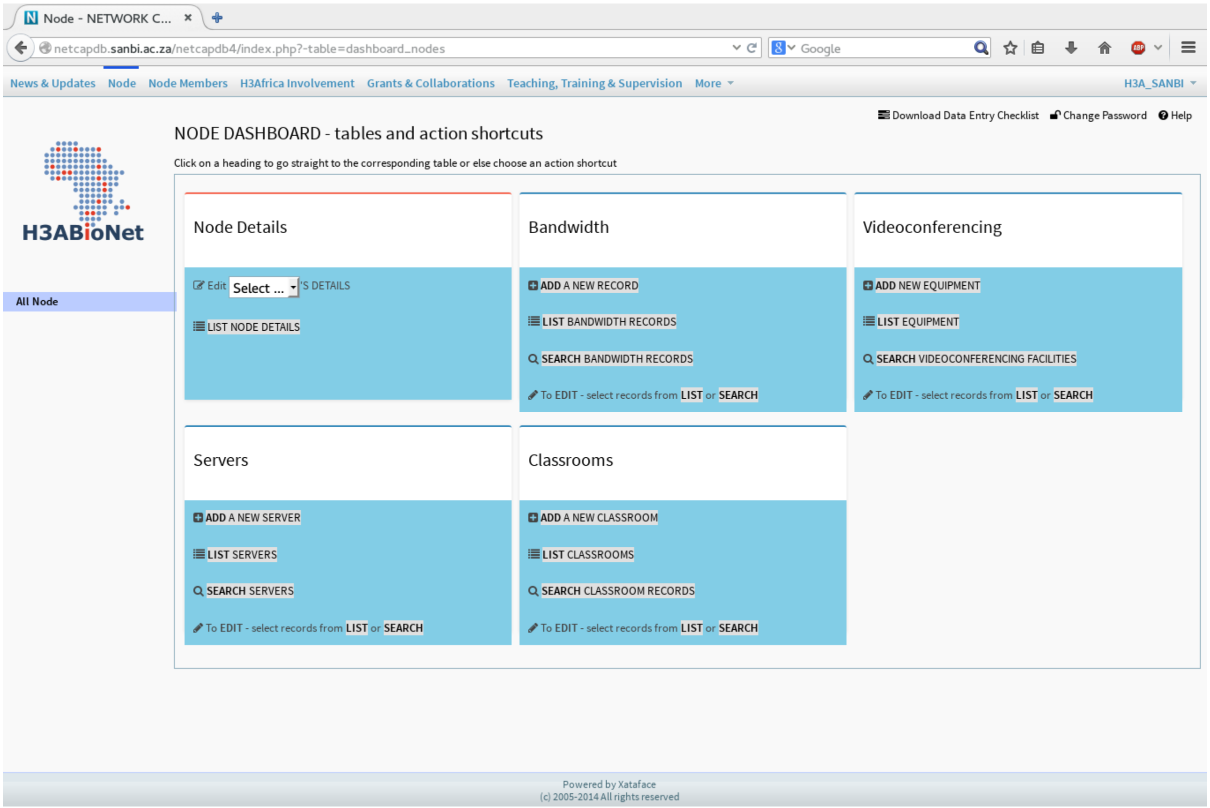

Figure 2: The *Node* dashboard.

First of all, and once only, every Node should fill in the “Node Details” table (institution acronym, institution name, physical address, etc) in this Node dashboard, in order to enable

us to identify properly the Node in question.

All other table from the same dashboard refer to non-static data, and so should be filled for every Reporting Period. Ideally, a personnel from the Node's IT staff should fill in these "Bandwidth", "Videoconferencing", "Servers" and "Classrooms" data tables.

2.2 Node Members dashboard

See figure 3.

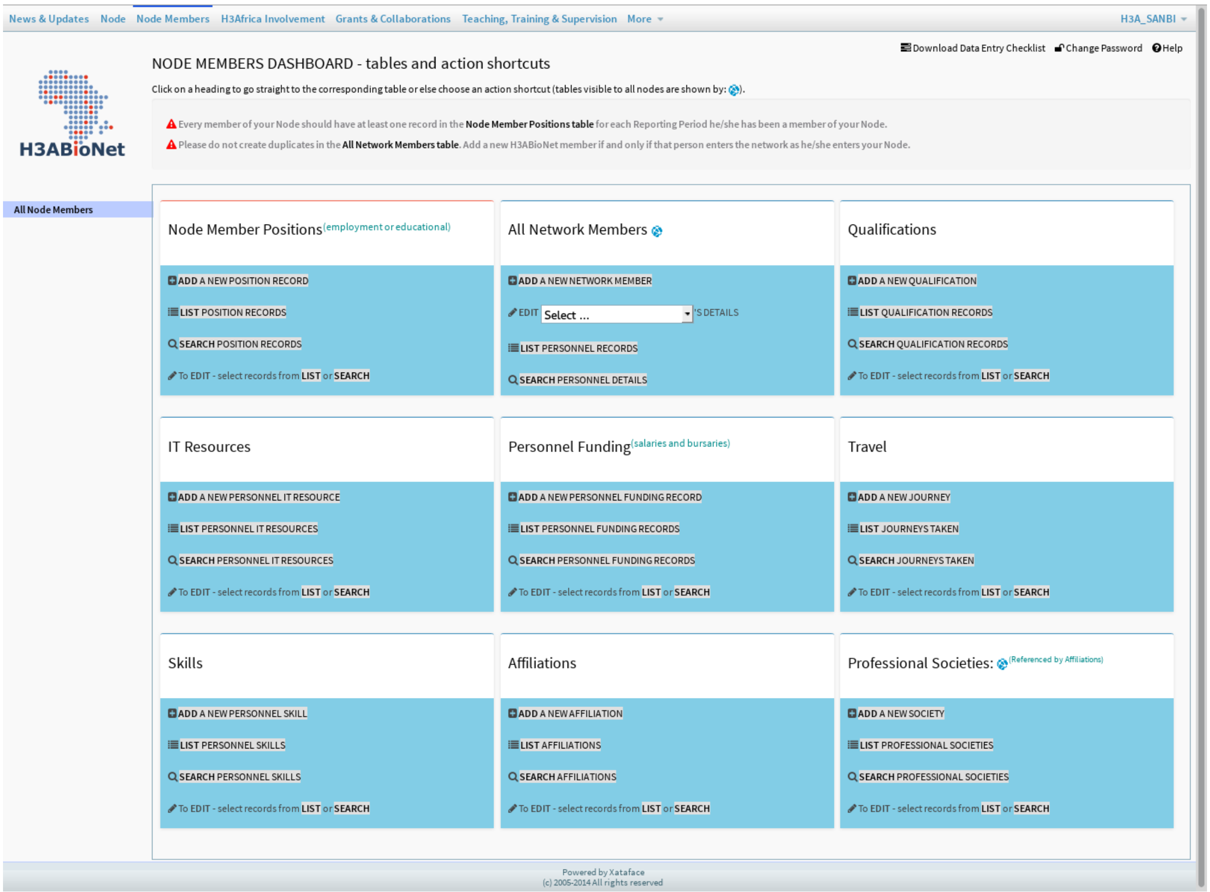

Figure 3: The *Node Member* dashboard.

2.2.1 All Network Members

There has been a major change in this dashboard: as we don't want the same person to be entered twice by different Nodes where he/she has been spending time, from now on, the table containing the H3ABioNet personnel identification details (full name, email address, citizenship, etc) has been made global, i.e. browsable and editable by all Nodes.

In that table of personnel details, please fill in your date of birth as an additional means of disambiguation between potential homonyms. You also have the possibility to enter your unique researcher identifier, if you have one (see `http://orcid.org/`). We strongly encourage

you to create your ORCID if you don't have one yet.

Also please pay attention to correctly input your first name (aka given name, forename, or "prénom" in French) in the right field, and your last name (aka surname, family name, or "nom de famille", "nom patronymique" in French) in the correct field as well, not swapping one for the other.

2.2.2 Node Member Positions

Again: we want no duplicates in the "All Network Members" table. If somebody joins your Node who was previously somewhere else in the H3ABioNet network, you will *not* enter him/her a second time in the "All Network Members" area. Instead, you have to create a record in the first table of the "Node Member" dashboard (called "Node Member positions") picking up the name of the right person in the drop-down list "H3ABioNet member" appearing in the form you get after having clicked on "Add a new position record".

Please pay attention to the fact that every member of your Node should have at least one record in the Node Member Positions table for each Reporting Period he/she has been a member of your Node.

In all the personnel-related records throughout the data entry tool, you will NOT be able to pick up your "Node Members" who have no record in the Node Member Positions table: the system simply doesn't recognize them as being part of your Node, even if you have initially entered their personnel details under the "All Network Members" area.

If at some point you see a "P0020" or something similar appearing in the list of records for some table instead of a full name, this is also a clue that you failed to fill in the "Node Member Positions" table for the corresponding Node Member.

2.2.3 Personnel Funding

This table is aimed at recording the information about how much of Node Member's timeshare is supported by such and such Funding Body. It is *not* the purpose of this table to record grants (for this, see "Grants" in the "Grants and Collaborations" dashboard) or any other money secured that you are using for other purposes than salaries or bursaries.

2.2.4 Travels

Please don't forget to fill in this table every time you travel outside of your Node for professional reasons (conference, collaboration, training, etc). Using the "Copy" button from the List view, you can record multiple travels to the same destination for the same reason, etc. during the same Reporting Period. This can be useful e.g. if you travel several times a year for the same collaborative project.

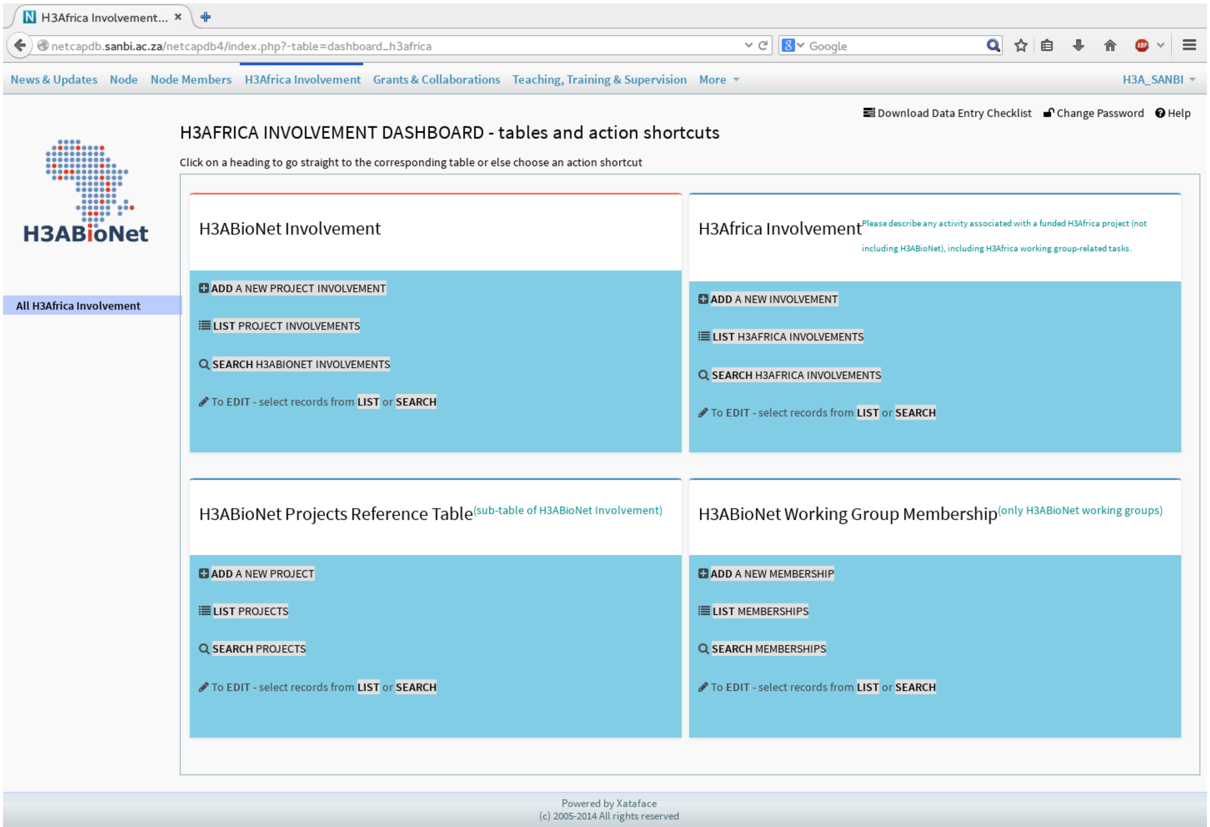

Figure 4: The *H3Africa Involvement* dashboard.

2.3 H3Africa Involvement dashboard

This dashboard contains four tables. The first one ("H3ABioNet Involvement", upper left) indicates which H3ABioNet project(s) a Node Member is working on during a given Reporting Period. This is essential to track down the implication of the various network members in the various projects funded by the network.

The table called "H3Africa Involvement" is meant to record all the H3ABioNet Members involved in H3Africa projects that are not H3ABioNet.

The "H3ABioNet Projects Reference" table is not a global one. It serves to keep track of the various projects one or several of your Node Members are involved in. The contents of this table derive from the "H3ABioNet Involvement" table.

Finally, in this dashboard, the "Working Group Membership" records the formal implication of your Node Members in the H3ABioNet Working Groups (e.g. Education and Training WG, Infrastructure WG, Research and Tool Development WG, etc).

2.4 Grants & Collaborations dashboard

See figure 5.

Nothing much to say here. Please pay careful attention to the currency you specify when

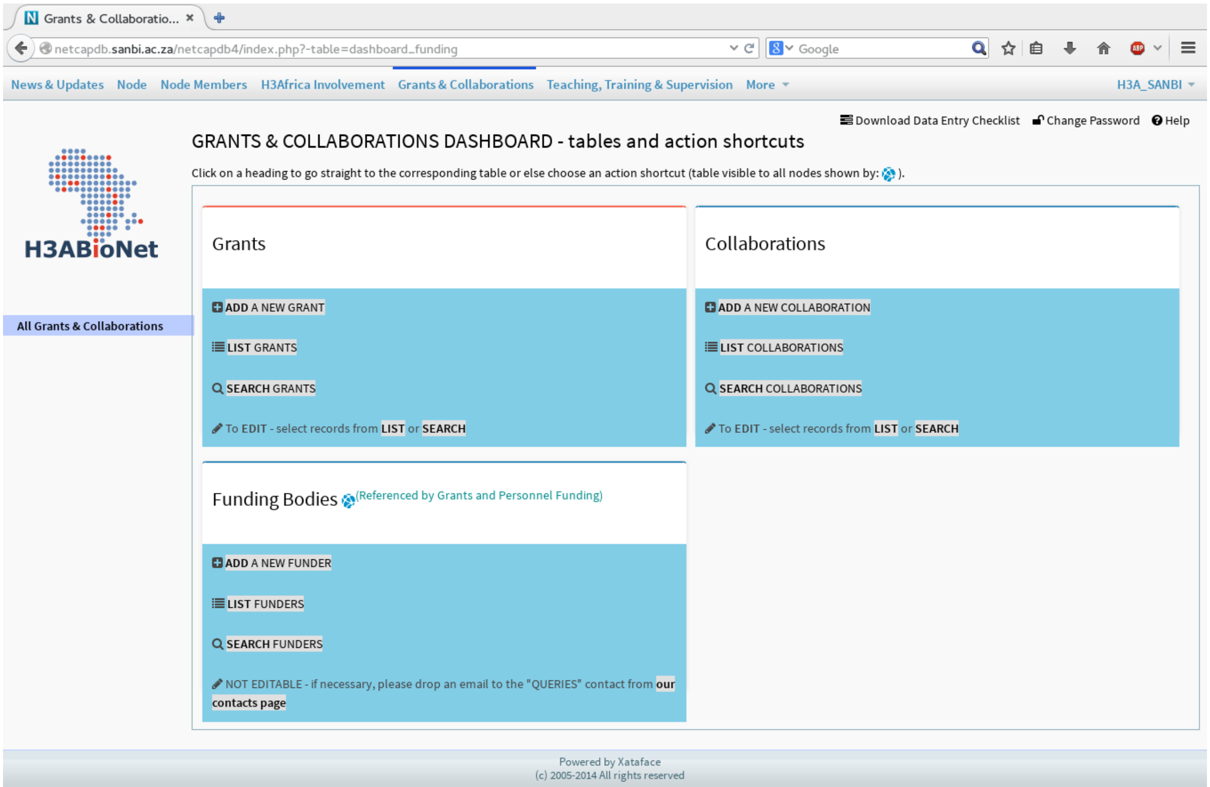

Figure 5: The *Grants & Collaborations* dashboard.

entering a grant. In the "Collaborations" table, please enter as many records as you have partners/sites in a specific collaboration (i.e. n-1 records for a collaboration over n research teams including yours). If you are working with a senior researcher and one of his/her students both working together in the same institution, please only enter one record for that collaboration.

Remember that once you have entered a record, you can use the "Copy" tool to add a new record deriving from that first one.

2.5 Teaching, Training & Supervision dashboard

This dashboard (figure 6) is also essential to NetCapDB. Every time a student from your Node attends a workshop or long course, every time a new student enters your Node under the supervision of an H3ABioNet member, that information should be recorded into the database. Of course, this means that all students working under the supervision of an H3ABioNet member should automatically be entered in NetCapDB. This is done first by creating his/her details in the "All Network Members" table under the Node Member dashboard, then by adding him/her as a Student during the relevant Reporting Period(s), through the "Node Member Positions" table under the same dashboard.

2.5.1 As a trainer

Here you report all the courses you have taught, be they part of a regular curriculum or of an H3ABioNet course/workshop.

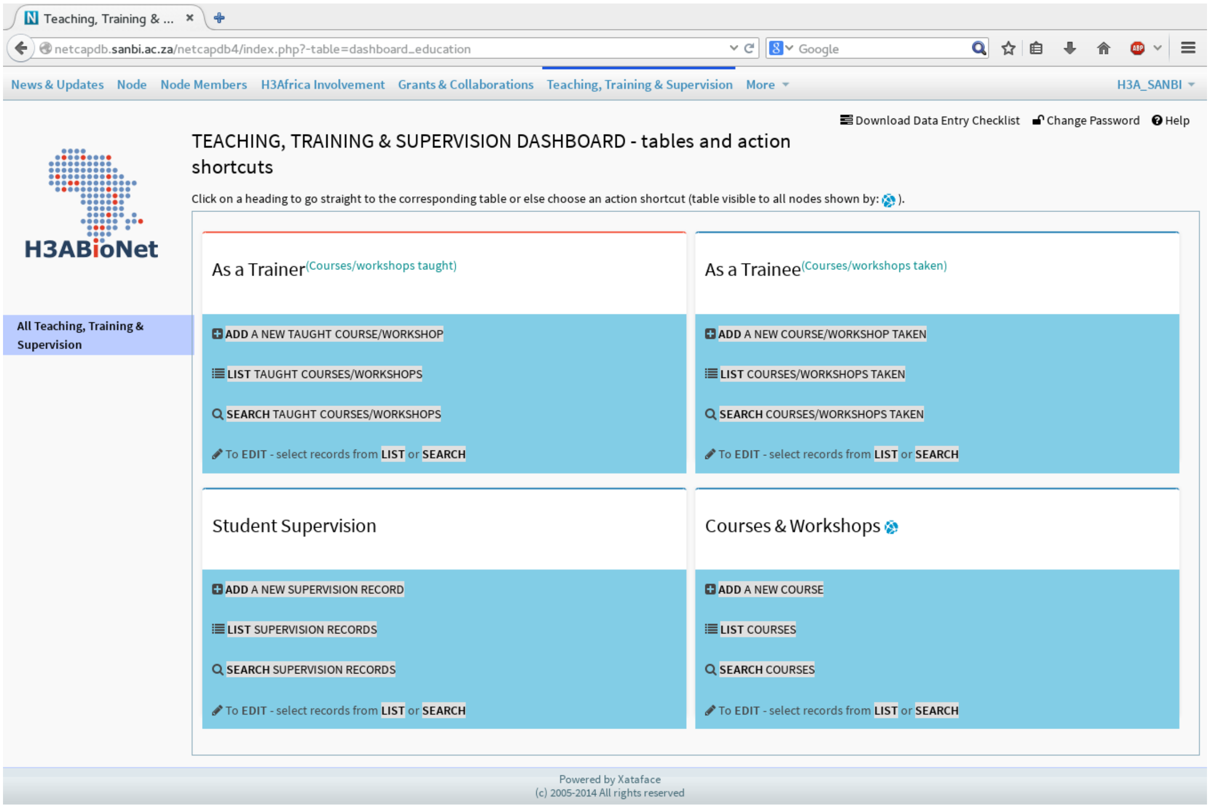

Figure 6: The *Teaching, Training & Supervision* dashboard.

2.5.2 As a trainee

This is the reciprocal of the previous table, where you report all the courses you have been taking over the course of a Reporting Period.

2.5.3 Student Supervision

Please indicate carefully whether the planned degree has been achieved during the course of the Reporting Period. Both supervisor and supervised should be in the database, so even if you are supervising somebody e.g. for a "sandwich PhD" and if that somebody is in Europe, you have to add that student in the "All Network Members" and in the "Node Member Positions" tables. Of course, we are talking about real supervision over the course of several months, not just counselling or reporting on somebody's thesis.

2.6 Publications & Conferences dashboard

This dashboard is shown in figure 7.

2.6.1 Publication Details

Most importantly, this table records all the publications by the members of your node. Publications include:

- articles published in peer-reviewed journals;
- books written or edited;
- book chapters contributed;
- articles featured in the proceedings of a conference;
- posters presented at a conference or workshop.

To enter publications in NetCapDB, we encourage you to use the PubMed bulk import tool, through the *Bulk import new publications* button from the *Publication Details* box. This will import a list of publications that you will have first downloaded to csv format from the PubMed website.

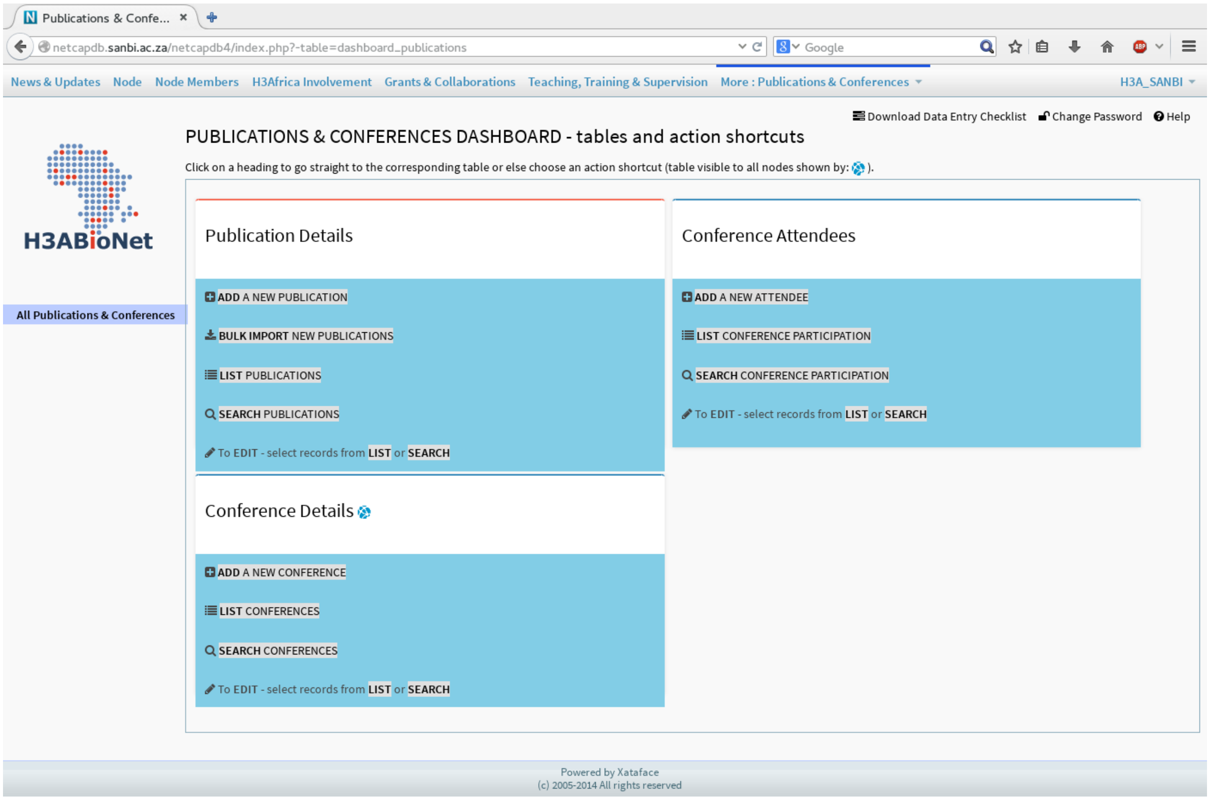

Figure 7: The *Publications & Conferences* dashboard.

You can also enter your publications one by one manually, using the *Add a new publication* button. In this case, please abide by the PubMed rule, which commands to write down an author's name spelling his/her last name first, followed by the concatenated initials of his/her given name(s) with no dots, e.g. « Stallman RM » for Richard Matthew Stallman or « Tiffin N » for Nicki Tiffin.

Please avoid duplicates: in case two or more co-authors of the same paper originate from your Node, they must obviously enter that publication only once.

2.6.2 Conference Attendees

Please record there the participation of any of your Node Members to a scientific conference. You are also invited to record whether the conference attendee has also given an invited/keynote talk, a short talk or presented a poster.

2.7 Reporting Periods dashboard

This last dashboard contains only one table, the global table of the Reporting Periods that make for the timespan of all events possibly recorded in the database. It is for consultation only and can not be modified. At the moment when we write this tutorial, the first Reporting Period is the 2007/2008 Reporting Period, and the last one is 2014/2015.

We encourage you to record data into NetCapDB as soon as the event (e.g. publication, new student supervision, conference attended, student graduated, etc) has happened, but please do not record in advance events that are planned, because it would be too easy to forget to delete the corresponding record in case that event would finally be cancelled or postponed.
